# Did the last common ancestor have a biological membrane?

**DOI:** 10.1186/1745-6150-1-35

**Published:** 2006-11-27

**Authors:** Gáspár Jékely

**Affiliations:** 1 European Molecular Biology Laboratory, Meyerhofstrasse 1, Heidelberg, Germany

## Abstract

All theories about the origin and evolution of membrane bound cells necessarily have to cope with the nature of the last common ancestor of cellular life. One of the most important aspect of this ancestor, whether it had a closed biological membrane or not, has recently been intensely debated. Having a consensus about it would be an important step towards an eventual (though probably still remote) synthesis of the best elements of the current multitude of cell evolution models. Here I analyse the structural and functional conservation of the few universally distributed proteins that were undoubtedly present in the last common ancestor and that carry out membrane-associated functions. These include the SecY subunit of the protein-conducting channel, the signal recognition particle, the signal recognition particle receptor, the signal peptidase, and the proton ATPase. The conserved structural and functional aspects of these proteins indicate that the last common ancestor was associated with a hydrophobic layer with two hydrophilic sides (an inside and an outside) that had a full-fledged and asymmetric protein insertion and translocation machinery and served as a permeability barrier for protons and other small molecules. It is difficult to escape the conclusion that the last common ancestor had a closed biological membrane from which all cellular membranes evolved.

## Background

The (near) universality of the genetic code and the universal presence in all sequenced genomes of key components of translation proved beyond any doubt that all cellular life on Earth derives from one common ancestor. Yet, beyond these universal features the nature of the last common ancestor of cellular life (or LUCA) is still intensely debated [1-5]. The views range from a non-membrane bound, minerally compartmentalised pre-cell [2-4,6,7] to a complex Gram-negative bacterium with a double membrane [5,8]. The universal presence of two transmembrane proteins, the F_0_F_1_-ATPase and SecY seems to suggest that the universal ancestor was a membrane bound cell [1]. However, this argument has recently been challenged by the proposition that proteins with transmembrane helices were not inserted into 'biological membranes' but into 'hydrophobic layers' of C8–C12 aliphatic acids [3]. In this scenario archaebacterial and eubacterial cells originated independently from a minerally compartmentalised common ancestor.

The idea of a membrane-less, minerally compartmentalised universal ancestor has been proposed because archaebacteria and eubacteria have membrane lipids of different chemical composition and chirality (archaebacteria have isoprenoid ethers of glycerol-1-phosphate, eubacteria have fatty acid esthers of glycerol-3-phosphate) and because these different lipids are synthesized by mostly non-homologous enzymes [1,2,8]. If one assumes that none of the two membrane forms could have evolved gradually from the other one or from a mixed membrane, the conclusion that eu- and archaebacterial membranes originated independently is inevitable. However, the divide between archaebacterial and eubacterial membranes may not be as deep as often imagined. The enzymes responsible for the chirality of the glycerol phosphate isomers (archaebacterial G1PHD and eubacterial G3PHD) also belong to larger enzyme families widely distributed among prokaryotes. G1PHD, synthesizing archaebacterial glycerol-1-phosphate, can even be found in Gram-positive bacteria [1]. Those authors who advocate a cellularised universal ancestor argue that eu- and archaebacterial membranes either evolved from heterochiral membranes [1], or by lipid phase segregation [9], or by the replacement of eubacterial lipids by archaebacterial ones due to adaptation to hyperthermophily [8].

Here I discuss what properties can we assign to the membranes or hydrophobic layers of the universal ancestor by carefully analysing the structural and function aspects of the universal membrane-associated cellular machineries.

## Discussion

### The universal ancestor had full-fledged membrane protein insertion and translocation machinery

In all cells the translocation of proteins across the plasmamembrane (or ER in eukaryotes) and the insertion of most transmembrane proteins are mediated by a transmembrane protein complex, the protein-conducting channel (PCC, SecYEG complex in eubacteria, Sec61 complex in eukaryotes) [10,11]. Proteins to be translocated carry an N-terminal signal sequence that is recognised by the signal recognition particle (SRP) as the preprotein emerges from the ribosome during translation. The SRP is targeted to the membrane via the SRP receptor where the signal peptide is transferred to the PCC, through which the protein is subsequently threaded (either cotranslationally or posttranslationally). The signal peptide is eventually cleaved by a serine protease, the signal peptidase, releasing the mature protein from the trans side of the membrane. Transmembrane proteins do not carry a cleavable signal peptide but their membrane insertion is mediated by hydrophobic membrane-spanning segments that are released into the membrane at the lateral side of the PCC.

Comparative genomic surveys revealed that the central components of the translation, protein insertion and translocation machineries are present in all forms of cellular life [12,13]. The ribosome, the SecY subunit of the PCC, the SRP54 GTPase that recognises the signal peptide, the SRP receptor FtsY/SRα, and the signal peptidase are universally conserved (SecE and SecG are not universal, but SecY can mediate translocation alone without these accessory subunits).

**Figure 1:**
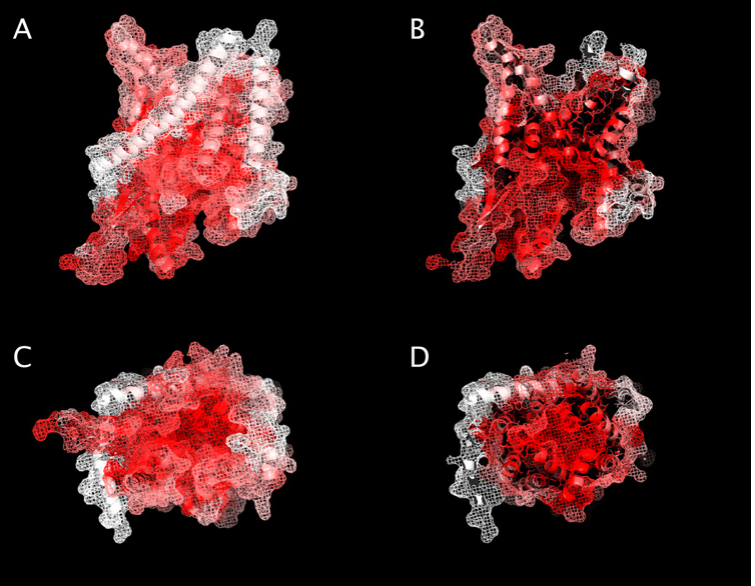
**Evolutionary conservation of the SecY channel**. Structure of the *Methanococcus jannaschii *SecY protein with sequence conservation mapped onto it. Conservation scores for SecY were calculated using the ConSurf server [22-24] based on a multiple alignment of 23 archaebacterial, 25 eukaryotic and 24 eubacterial SecY sequences. The conservation scores were displayed on the structure of SecY from *Methanococcus jannaschii *[15] (PDB code 1RH5) using Pymol [25] and the color_b.py script [26]. A) and B) are lateral views from the plane of the membrane, C) and D) are cytoplasmic views. B) and D) are sectioned at the middle of the molecule.

The conserved topology and sequence features of SecY reveal that the PCC is ancestrally membrane associated. SecY has an extracellular and an intracellular hydrophilic part, a conserved inner pore and ten conserved transmembrane segments [14,15] (Fig. 1). The universality of this arrangement indicates that the hydrophobic layers of the universal ancestor into which SecY was integrated had a hydrophobic core and two hydrophilic sides.

In SecY the binding pocket of N-terminal signal sequences is also universally conserved and is situated in the lumen of the channel [15] (Fig. 1). This conservation and the similarity of N-terminal signal peptides in all cells [16] indicate that not only the PCC, but also signal sequences to bind to it, were present in the last common ancestor. Signal peptides after translocation are cleaved by the universal and essential signal peptidase [17,18]. This serine protease is located on the extracellular (or periplasmic) side of the plasmamembrane and has an N-terminal transmembrane helix that anchors the catalytic domain to the membrane.

There is a clear asymmetry in the arrangement of the secretory machinery: signal peptide containing proteins bind to the PCC at one side, are translocated across the PCC, and are cleaved at the other side by the signal peptidase. The asymmetric arrangement of this machinery is self-maintained: the asymmetric SecY channel inserts new copies of itself and translocates new copies of the signal peptidase to the outside. This asymmetric arrangement is universally conserved indicating that it already existed in the universal ancestor. This means that the hydrophobic layers of the universal ancestor were asymmetric with an 'inside' and an 'outside' and this asymmetry was maintained autocatalytically. The active translocation of secreted proteins by the ribosome-signal peptide-SRP-PCC machinery also means that the hydrophobic layers of the universal ancestor must have presented a permeability barrier (i.e. were closed). Otherwise where and why would signal-peptide containing proteins have been translocated?

### The universal ancestor had a membrane that provided permeability barrier

There is additional evidence that the universal ancestor had a closed membrane that presented a permeability barrier to small molecules. As shown by comparative genomics, the last common ancestor carried in its membrane the ancestor of the universal membrane protein complex, the F_0_F_1_-ATPase. F_0_F_1_-ATPases generate a transmembrane electrochemical gradient at the expense of ATP or catalyze the synthesis of ATP using an electrochemical gradient [19]. Although the exact composition of the complex cannot be inferred because it has many non-related subunits in archaebacteria and eubacteria, its ancestral presence can safely be established. The universal distribution of the proton translocating F_0 _c subunit of the complex and the conserved function of the proton ATPase indicate that the last common ancestor was associated with a hydrophobic layer able to maintain a proton gradient, i.e. it was necessarily closed. A hydrophobic layer by itself, if not closed, is not enough for proton ATPase function. Importantly, the PCC also uses a proton gradient to drive the insertion of transmembrane proteins [20] highlighting the importance of a closed membrane for transmembrane protein insertion.

## Conclusion

**Figure 2:**
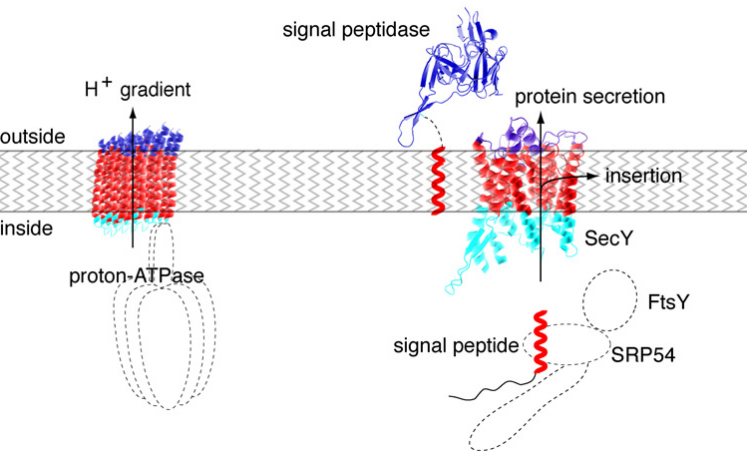
**Reconstruction of some aspects of the last universal ancestor's membrane**. The hydrophobic segments of secretory proteins, SecY (PDB code 1RH5), the c subunit of the F_0 _ATPase (PDB code 1wu0), and the signal peptidase (PDB code 1B12) are in red. Extracellular segments are in blue, cytoplasmic segments in cyan, as defined in the PDBTM database [27]. The c subunit of the F_0 _ATPase is shown in ten copies, as is found in the *E. coli *complex [19].

By the comparative genomic and functional reconstruction of membrane-associated functions the following characteristics can be attributed to the hydrophobic layers of the last universal ancestor. These layers had a (i) hydrophobic core with two hydrophilic sides (ii) had a protein insertion and translocation machinery (iii) had a clear asymmetry (iiii) represented a permeability barrier to proteins and small molecules (see Figure 2). It is difficult to escape the conclusion that this was a closed membrane of a membrane-bound cell from which all biological membranes evolved.

The above arguments were based on a very conservative reconstruction taking into account only universally distributed components. More recent reconstructions also allowing for extensive gene losses give much higher (about 1000) numbers for the gene content of the universal ancestor [21]. If this is anywhere close to the truth then a membrane-less universal ancestor loses all its credibility. It is also to be considered that the membrane-less universal ancestor model [3] as well as the above discussion is based on the unproven assumption that the tree of life is rooted between archaebacteria and eubacteria. If it is not the case [5], the argument is again futile.

Not accepting the cellular nature of the last common ancestor would mean that membrane-bound cells originated twice independently. The origin of cellular life where genes, membranes and catalysts are integrated in a replicating whole is probably the most difficult problem in cell evolution. The theory of the independent origin of eu- and archaebacterial cells has to solve this problem twice and therefore is clearly most unparsimonious. Given the evidence summarised here it is hard not to conclude that the universal ancestor was membrane bound. In this case it is time to start seriously considering different scenarios of lipid segregation or replacement [1,8,9] to account for the different lipid composition and chirality of archaebacterial and eubacterial membranes.

## Competing interests

The author(s) declares that he has no competing interests.
